# A case report of interventricular hemorrhage in William-Beuren syndrome

**DOI:** 10.1016/j.amsu.2022.104305

**Published:** 2022-08-05

**Authors:** Abdullah Alghobaishi, Ahmed Hafez Mousa, Haleema Sami Almonaye, Tasneem Khalid Maghrebi, Abeer Amin, Fawziah Alzaid Al Sharif

**Affiliations:** a Department of Pediatrics, Pediatrics Critical Care Medicine, King Fahad Armed Forces Hospital, Jeddah, Saudi Arabia; b College of Medicine and Surgery, Batterjee Medical College, Jeddah, Saudi Arabia; c Department of Pediatrics, Faculty of Medicine, Cairo University, Cairo, Egypt; d Department of Pediatrics, Saudi German Hospital, Jeddah, Saudi Arabia; e Children's Health Center, Department of Pediatrics, International Medical Center, Jeddah, Saudi Arabia

**Keywords:** Williams syndrome, Williams–beuren-szindróma, Genetic analysis, 7q11.23 deletion

## Abstract

**Background:**

Williams syndrome (WS) (also as known Williams-Beuren Syndrome) is a neurodevelopmental disorder caused by deletion of chromosomes 7q11.23. WS phenotype is very variable but usually it is associated with a distinctive pattern of cognitive abilities.

**Case presentation:**

9-year-old female patient known case of developmental delay and precocious puberty presented to the emergency department with altered level of consciousness, elevated blood pressure of 230/160 with a provisional diagnosis of hypertensive encephalopathy.

**Conclusions:**

In our patient, the strongest indications for establishing the diagnosis included the classic elfin face and multisystemic involvement. Later on genetic analysis confirmed our diagnosis.

## Highlights

• Williams syndrome (WS) (also known Williams-Beuren Syndrome) is a neurodevelopmental disorder caused by deletion of chromosomes 7q11.23
• We recommend further studies to investigate the complexity of cardiac and vascular manifestations associated with WS. Particular focus on the managment of the severe hypertension associated is recommended.
• In our patient, the strongest indications for establishing the diagnosis included the classic elfin face and multisystemic involvement.

## 1 Background

Williams syndrome (WS) (also known Williams-Beuren Syndrome) is a neurodevelopmental disorder caused by deletion of chromosomes 7q11.23 [1]. This syndrome has a potential impact on all organ systems [2]. Classic findings include but are not limited to, a distinct elf-like face, cardiovascular abnormalities, growth retardation, connective tissue abnormalities and endocrine abnormalities [3]. WS phenotype is very variable but usually it is associated with a distinctive pattern of cognitive abilities [4]. Studies have reported that the IQ of these patients’ range between 40 and 90 [5]. The vast majority of WS cases diagnosed on clinical basis have demonstrated deletion of chromosomes 7q11.23 that has been detected by FISH (fluorescent in situ hybridization) [6]. Gene mapping has been capable of detecting various elastin mutations that have been attributed to cardiovascular manifestations of WS as isolated supravalvular aortic stenosis [7]. The prevalence of WS is estimated to be 1 in 7500 [8]. This case has been reported in accordance with the SCARE criteria [17].

## 2 Case presentation

This is a case of a 9-year-old female patient known case of developmental delay and precocious puberty presented to the emergency department with altered level of consciousness, elevated blood pressure of 230/160 with a provisional diagnosis of hypertensive encephalopathy.

Serum calcium, sodium and potassium were normal. However, urea level was 35.10 and creatinine was 1.48 and GFR40 ml/min 1.73M2. Complete blood count was normal other than hemoglobin level of 9.32 and platelet count of 285,000. Brain MRI with MRA and MRV revealed an intraparenchymal hemorrhage in the left basal ganglia, acute intraparenchymal hematoma involving the right basal ganglia associated with edema, effacement of the overlying cortical sulci and mass effect with compression on the adjoining parts of the right lateral ventricles. Inter ventricular hemorrhage and dilation predominantly affecting right lateral ventricles, and subarachnoid hemorrhage. Brain CT without contrast confirmed MRI findings and showed mild to moderate acute hydrocephalus and features of increased intracranial pressure CT chest, abdomen and pelvis aortic angiography showed global left ventricular myocardial hypertrophy, dilation of pulmonary arterial tree, subtle diffuse uniform descending aortic mural thickening due to severe long-standing hypertension, and mild mural thickening of Jejunal and proximal ileal loops. Detailed nephrological assessment revealed grade II-III bilateral nephropathy renal failure with echogenic kidney and no renal artery stenosis. Initially she was started on labetalol 15mg Q6h to be held if SBP<130 mm hg and Bedside labetalol 1mg/kg/hr and labetalol infusion and hydralazine 20 mg/ml PRN and IVF D5 Ns 60%M. her blood pressure improved ranging 130–140 systolic. A day after admission to the PICU, she developed features of left facial palsy on smiling or crying, with facial deviation to the right side, bilateral edema of eyelids and urine output was 4.1ml/kg/hr which progressed to 1.1 ml/kg/hr. Because her blood pressure did not reach target, amlodipine 2.5mg/day and enalapril 2.5mg/day were added to control hypertension. Her blood pressure kept rising so labetalol infusion increased to 2mg/kg/hr, labetalol increased to 20mg every 6 hours enalapril to 5mg twice daily instead of once daily and amlodipine 5mg once daily. Yet still her blood pressure ranged 140–150 systolic so enalapril increase to 7.5mg twice daily. A trial to wean labetalol infusion to 0.5 mg/kg/hr was done, but due to high readings, it increased back to 1mg/kg/hr and hydralazine 15 mg every 6 hours regular started, and labetalol PRN Q 4h IV Lastly, Enalapril dose increased to 10 mg twice daily, Amlodipine to 5 mg OD. A karyotype sample was requested to confirm the diagnosis. The sample obtained was heparin blood and 30 cells were analyzed. Karyotype (ISCN2020): ish del (7) (q11.23q11.23) (ELN/LIMK1/D7A613-). The signals of the probes were found only on one of the chromosomes which were identified as chromosomes 7 by simultaneous hybridization of probe D7S486/D7S522 (7q31). These findings indicate a deletion in the Willams-Beuren

**Fig. 1:**
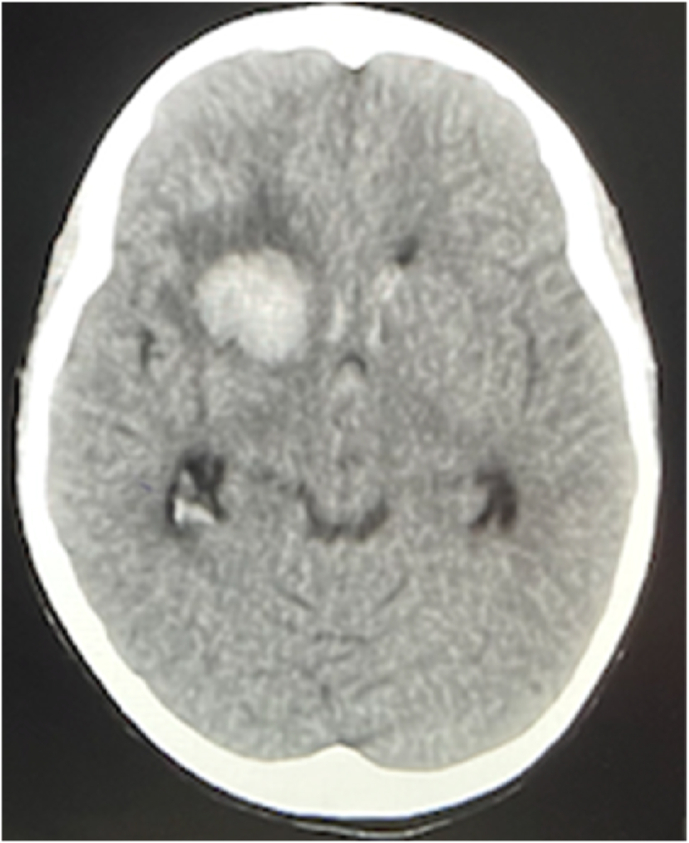
Non-contrast CT study shows right sided basal ganglia hyperdense hemorrhage noted with mass effect ipsilateral lateral ventricle and related perifocal edema. Extension of the hemorrhage into the ventricular system.

**Fig. 2:**
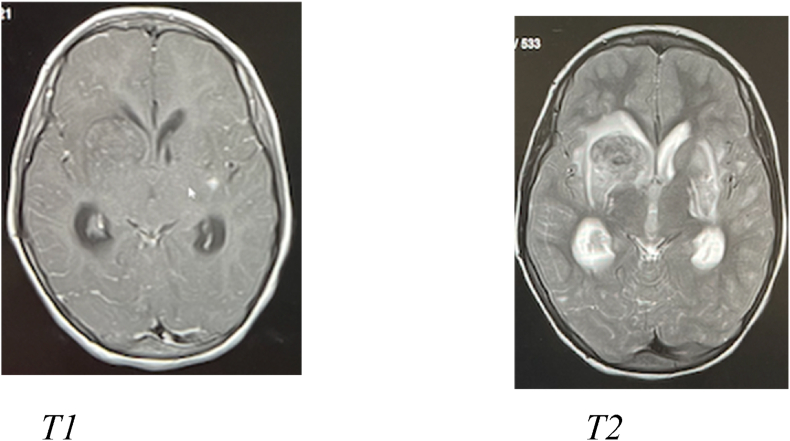
MRI study shows the hemorrhage to be isodense and intense in T1 and T2. However, it shows blooming of the gradient sequence confirming the ipsihemorrhagic nature also there are left basal ganglia hemorrhage noted in the gradient sequence. CTA of the aorta reveals mild narrowing in the distal aorta bimural thickening and apparent hypertrophy of the left ventricular wall.

Syndrome critical region in one of the 7 chromosomes, thereby conforming the suspected diagnosis. Fig. 1, Fig. 2 shown below demonstrate the most significant brain findings.

## 3 Discussion

Patients with WBS are at risk for systemic hypertension mainly due to congenital heart defects that are present 75% of patients [9]. However, this case of WBS came in with hypertensive encephalopathy with IVH of unidentifiable cause, a rare outcome in these patients. Echocardiography of our patient revealed descending aortic mural thickening and severe left ventricular hypertrophy, denoting long standing hypertension, in addition, changes in pulmonary vasculature like dilation of pulmonary arterial tree was noted. Other lesion common of WBS like aortic stenosis or aortic coarctation were not present. Supravalvular aortic stenosis, a lesion that is found in 70% of patients, is rare except in WBS and familial supravalvular aortic stenosis syndrome [10]. The prevalence of hypertension in WBS in children below the age of 16 ranges up to 17%, but a clear etiology is only found in few [[10], [11]]. Other cardiovascular lesion of this syndrome that can contribute to hypertension is renal artery stenosis, which usually occurs in 7–58% of patients [12]. But our patient's CT angiography of renal arteries showed no abnormalities. Her kidney function tests revealed grade 3 nephropathy kidney failure which we attributed to her hypertension. The true incidence of renal anomalies in WBS is unknown, some literature estimates it to be 17–18%. The spectrum of renal anomalies ranges from bladder diverticula and urinary tract infections to renal hypoplasia, vesicoureteral reflux, and renal artery stenosis [13]. Thus, ultrasound screening should be part of evaluation of WBS patients.

Our patients' diagnosis was suspected based on her typical dysmorphic features and intellectual delay, other features present is periorbital puffiness, based on a study done on diverse populations with WBS found this feature to be present in 90% of subjects [14]. Endocrinal abnormalities in WBS include early onset puberty/precocious puberty, diabetes mellitus, osteoporosis/osteopenia, subclinical hypothyroidism, and hypercalcemia [15]. Assessment of thyroid function tests is recommended at time of diagnosis of WBS, annually for first three years of life, and every 2 years after. Our patient's thyroid function tests were within normal range, a regular assessment is recommended as mentioned. Adults with WBS have an increased prevalence of type 2 diabetes mellitus, consequently oral glucose tolerance test should be done at 30 years and repeated every 5 years [16]. True precocious puberty is another rare finding that our patient is present with.

Management of WBS relies on symptomatic treatment of manifestations the patient is present with. Our case presented with hypertensive encephalopathy with blood pressure readings of 230 systolic and 160 diastolic pressures at admission and was immediately started on labetalol for management. Her blood pressure readings with labetalol infusion 5 days after admission read a systolic pressure of 141–157-161 and diastolic pressure of 99–96-112 at 1st, 12th, and 23rd hours respectively. Her response to treatment seems to be minimal with beta blockers with fluctuations still present. Initial approach for patients with HTN is investigation for possible vascular injuries that can be surgically corrected such as renal artery stenosis or supravalvular aortic stenosis both of which were absent in this patient. Others would recommend starting with antihypertensive medications before searching for vascular cause. Calcium channel blockers like amlodipine or nifedipine are the first line therapy for such patients. If blood pressure cannot be controlled, beta-blockers as labetalol can be added. Angiotensin-converting enzyme inhibitors and diuretics can be used for hypertension refractory to both calcium channel blocker and beta blockers [17]. A multidisciplinary approach with a team involving an endocrinologist, a neurologist, a nephrologist, a neurosurgeon, and an interventional radiologist was initiated for our patient. Her interventricular hemorrhage resolved after 2 months without intervention despite her persistent hypertension. Following her stabilization, she was discharged home with an outpatient follow up.

## 4 Conclusions

We suggest that more evaluation of the cardiac involvement should be performed especially when a patient has abnormal facial features. In our patient, the strongest indications for establishing the diagnosis included the classic elfin face and multisystemic involvement. Later on diagnosis was confirmed by genetic analysis. Labetalol, amlodipine and enalapril were very essential in the management of the hypertension associated with this condition.

## Ethical approval

Ethical approval has been given by the Institutional Review Board (IRB) of our institution, Saudi German Hospital, Jeddah, Saudi Arabia.

## Sources funding

No funding for this research.

## Author contributions

Drafting of the manuscript: Ahmed Hafez Mousa, Haleema Sami Almonaye, Tasneem Khalid Maghrebi, Abeer Amin, Fawziah Alzaid Al Sharif, Abdullah Alghobaishi.

Critical revision of the manuscript for important intellectual content:

Ahmed Hafez Mousa, Haleema Sami Almonaye, Tasneem Khalid Maghrebi, Abeer Amin, Fawziah Alzaid Al Sharif, Abdullah Alghobaishi.

## Trial register number

1. Name of the registry:
2. Unique Identifying number or registration ID:
3. Hyperlink to your specific registration (must be publicly accessible and will be checked):

## Consent

Written informed consent was obtained from the patient for publication of this case report and accompanying images. A copy of the written consent is available for review by the Editor-in-Chief of this journal on request.

## Guarantor

Ahmed Hafez Mousa, corresponding author of the manuscript, accept full responsibility for the work and the conduct of the study, had access to the data, and controlled the decision to publish.

## Provenance and peer review

Not commissioned, externally peer reviewed.

## Informed consent

An informed consent has been obtained from the child's parents.

## Declaration of competing interest

No conflicts of interest.

## Abbreviations

WS –: Williams Syndrome
IQ –: Intelligence Quotient
GFR –: Glomerular Filtration Rate
BID –: Twice Per Day
PRN –: Parenteral Nutrition
OD –: Taken Once Everyday
WBS –: Williams Beuren Syndrome
